# Two-Stage Exchange Arthroplasty for Periprosthetic Shoulder Infection Is Associated with High Rate of Failure to Reimplant and Mortality

**DOI:** 10.3390/jcm10215186

**Published:** 2021-11-06

**Authors:** Doruk Akgün, Mats Wiethölter, Nina Maziak, Alp Paksoy, Daniel Karczewski, Markus Scheibel, Philipp Moroder

**Affiliations:** Center for Musculoskeletal Surgery, Charité-Universitätsmedizin Berlind, Charitéplatz 1, 10117 Berlin, Germany; mats-jonas.wiethoelter@charite.de (M.W.); nina.maziak@charite.de (N.M.); paksoyalp97@gmail.com (A.P.); daniel.karczewski@charite.de (D.K.); markus.scheibel@charite.de (M.S.); philipp.moroder@charite.de (P.M.)

**Keywords:** periprosthetic shoulder infection, two-stage exchange, mortality, reimplantation

## Abstract

Background: Patients with a periprosthetic joint infection (PJI) of the shoulder, who fail to undergo reimplantation in an attempted two-stage exchange seem to be neglected in the current literature. The aim of this study was to assess the clinical course of patients after the first stage in the process of an attempted two-stage exchange for shoulder PJI. Methods: After a retrospective review of our institutional database between 2008 and 2018, 49 patients, who were treated with an intended two-stage exchange for shoulder PJI, were identified. Patients’ demographics, laboratory and health status parameters, along with records of clinical outcome were collected. The primary outcome measurements analyzed were infection eradication, successful reimplantation, and patient survival. Results: Reimplantation was completed in only 35 (71%) of 49 cases and eradication of infection was achieved in 85.7% of patients with successful reimplantation after a mean follow-up duration of 5.1 years (1.1 to 10.2 years). Reasons for failure to reimplant were premature death in 36%, high general morbidity in 29%, satisfaction with the current status in 21%, or severe infection with poor bone and soft tissues in 14% of the patients. Of the 14 cases without reimplantation, eradication rate of infection was 57% after a mean follow-up of 5 years (2.6 to 11 years). The overall mortality rate of the entire cohort was 25% at the latest follow-up and 10% within ninety days after implant removal. Patients who deceased or did not undergo reimplantation during the follow-up were significantly older and had a significantly higher Charlson comorbidity index (CCI). Conclusions: While the two-stage exchange arthroplasty can lead to high rates of infection eradication, a considerable subset of patients never undergoes the second stage for a variety of reasons. Shoulder PJI and its treatment are associated with a high risk of mortality, especially in patients with older age and higher CCI.

## 1. Introduction

Periprosthetic joint infection (PJI) of the shoulder represents a devastating complication and is the main cause of revision within the first few years after shoulder arthroplasty [1,2,3]. Its treatment continues to pose a challenge for the orthopedic community [3,4]. Although the preferred surgical treatment of chronic shoulder PJI is still unknown and pooled data demonstrate single-stage exchange may be superior to two-stage exchange in selected cases, two-stage exchange arthroplasty with implant removal, insertion of an antibiotic spacer, followed by reimplantation of an arthroplasty, continues to be the most common treatment strategy for shoulder PJI [5,6]. The reported infection eradication rate of two-stage exchange arthroplasty varies in literature between 63% and 100% [4,6]. However, the majority of these studies focus on the clinical outcomes after successful reimplantation and overlook a substantial number of patients who undergo resection arthroplasty alone and do not complete the second stage of an attempted two-stage exchange arthroplasty [7,8]. Thus, these studies may not accurately reflect the overall success rate of two-stage exchange arthroplasty for shoulder PJI. Furthermore, most studies are limited to small case series and most importantly, there is a lack of data and high variability of how these studies define diagnosis and treatment success of shoulder PJI, which could result in an overestimation of the outcome parameters. An improved understanding of the interstage period and application of a standardized, multidimensional definition of shoulder PJI diagnosis and also treatment success is crucial to accurately depict the clinical outcome of two-stage exchange arthroplasty.

The purpose of the current study was to assess the clinical course of patients after implant removal in the process of an attempted two-stage exchange arthroplasty for shoulder PJI including infection eradication, successful reimplantation, and patient survival as main outcome parameters.

## 2. Materials and Methods

### 2.1. Study Design and Cohort

We conducted a retrospective analysis of all patients who were scheduled for a two-stage exchange arthroplasty in our institution between 2008 and 2018 due to a shoulder PJI. A total of 49 patients were identified from our prospectively collected institutional database and were included in the study. The study protocol was reviewed and approved by the institutional ethics committee (EA4/040/14).

The mean age of the patients at the time of the first stage of the two-stage exchange arthroplasty was 70 ± 11 years (range: 37–88 years) and 30 patients (61%) were female. The main reasons for primary shoulder arthroplasty were cuff arthropathy (10 patients), primary osteoarthritis (17 patients), fracture (19 patients), or posttraumatic osteoarthritis (3 patients). A total of 10 patients (20.4%) had undergone at least one previous septic revision in another hospital, 10 (20.4%) had undergone at least one aseptic revision, and 29 (59%) had developed a shoulder PJI after the initial arthroplasty. The type of arthroplasty at the time of the first stage revision surgery was hemiarthroplasty in 17 (35%), anatomic total shoulder arthroplasty in 9 (18%), and reverse shoulder arthroplasty in 23 patients (47%). The mean interval between the primary arthroplasty and implant removal surgery at our institution was 4.1 ± 3.7 years.

### 2.2. Data Collection

Comorbidities, history of the infected shoulder arthroplasty, the score of the Charlson comorbidity index (CCI) [9], laboratory values including serum C-reactive protein (CRP), and serum leucocyte count were recorded on admission. In addition, the following data were extracted for all patients: leucocyte count, microbiological and histopathological results of aspiration, number of revision surgeries between stages, length of interval between explantation and reimplantation, and microbiological and histopathological results of all surgeries. Furthermore, component loosening was evaluated radiologically and intraoperatively and documented in our database for every patient, as well as intraoperative findings such as cloudy fluid or gross intra-articular purulence. Patients were seen in our outpatient clinic postoperatively after 3, 6, and 12 months and after that period, annually. Clinical and radiological evaluations were performed by an orthopedic surgeon and infectious disease specialist. A standardized questionnaire evaluating the general health, joint and skin status, any additional surgical interventions, and antibiotic use was performed. Further follow-up was performed, contacting the patients by phone or during the visit in our outpatient clinic. The primary outcome measurements analyzed were treatment success in terms of infection eradication, successful reimplantation, and patient survival.

### 2.3. Definitions

Periprosthetic shoulder infection was diagnosed according to the last proposed definition criteria of the ICM [10]. According to these criteria, patients were classified into 4 infection subgroups: (1) definitive infection; (2) probable infection; (3) possible infection; (4) infection unlikely. Meeting one of the following criteria was diagnostic of definitive periprosthetic shoulder infection: (1) a sinus tract communicating with the prosthesis; (2) gross intra-articular pus; (3) two positive cultures with phenotypically identical virulent organisms. In the lack of these defining signs, weighted minor criteria (Table 1) are summed and used to distinguish between probable, possible, and unlikely infection.

The three categories in these less distinct scenarios are defined as follows:

- Six or greater with identified organism: probable infection.
- Six or greater without identified organism; possible infection.
- Fewer than six.
  - single positive culture virulent organism: possible infection.
  - two positive cultures low-virulence organism: possible infection.
  - negative cultures or only single positive culture for low virulent organism: infection unlikely.

Of the 49 infected patients, 16 met the criteria for definitive infection, 25 for probable infection, and 8 for possible infection. Cutibacterium acnes was the most common infecting microorganism at the time of resection arthroplasty in 18/49 patients (37%), followed by coagulase-negative staphylococci (18/49, 37%), Staphylococcus aureus (8/49, 16%), and other microorganisms (9/49, 18%). In 14 of 49 cases (29%), a polymicrobial infection was evident and eight patients (16%) had no growth in the microbiology. Three of these eight patients with negative microbiology had a definitive infection due to presence of gross intraarticular pus and antibiotic treatment was started before taking samples, as patients were in sepsis. In the remaining five patients, the infection was classified as possible due to presence of minor criteria.

The definition for successfully treated shoulder PJI, in terms of infection eradication, was based on the Dephi-based international multidisciplinary consensus [11] and was further modified [12,13]. Infection was considered as eradicated if all of the following criteria were fulfilled at the latest follow-up: infection eradication, characterized by a healed wound without fistula and drainage; no recurrence of the infection; no occurrence of periprosthetic joint infection-related mortality; no subsequent surgical intervention for infection after reimplantation surgery; no long-term (>6 months) antimicrobial suppression therapy. Given that the Delphi criteria do not consider patients who do not undergo the reimplantation stage of the two-stage exchange arthroplasty, in this study successful infection eradication also included no subsequent surgical intervention for infection after explantation and no mortality related to infection in patients who did not undergo the reimplantation stage [8].

### 2.4. Two-Stage Exchange Arthroplasty Approach

All patients were treated according to a standardized two-stage exchange protocol. The first stage consisted of removal of all implants, as well as infected tissue, cement, and all other foreign material followed by irrigation and debridement. In most cases an antibiotic-impregnated cement spacer was inserted. Tissue cultures were incubated for 14 days. Antibiotic treatment was started intravenously (IV) after surgery or preoperatively in the case of patients presenting with sepsis after synovial aspiration. A standardized antimicrobial treatment was applied in every case based on a previously published concept under the surveillance of our infectious disease specialists [14]. A revision with irrigation, debridement, and concomitant spacer exchange was performed in case of a persistent infection (discharging wound and/or local sings of infection and/or increasing CRP without any other focus). A reimplantation was performed when the operation site was healed, with soft tissues in a good condition and ready for surgery, and the general health status of the patient was suitable for surgery. The reimplantation was used as another chance to execute another debridement of the surrounding soft tissues and bone before reimplantation of the definitive components. Intravenous antibiotic treatment was given for 2 weeks after reimplantation surgery and changed to oral regime mostly for another 4 weeks to complete a total treatment duration of 6 weeks after reimplantation.

### 2.5. Statistical Analysis

Chi-squared and Fisher’s exact tests were used to find significant differences between categorical variables. The Kolmogorov–Smirnov test was used to test for normal distribution. The 2-sample *t* test (for parametric distributions) or Mann–Whitney U test (for nonparametric distributions) was used to compare continuous variables between groups. The results were given as the mean and standard deviation or as the number and percentage. For statistical analyses, IBM SPSS Statistics software (version 25.0; IBM, Armonk, NY, USA) was used. *p* < 0.05 was considered statistically significant.

## 3. Results

Reimplantation was completed in only 35 (71%) of 49 cases and eradication of infection was achieved in 85.7% of patients with successful reimplantation after a mean follow-up duration of 5.1 years (range: 1.1 to 10.2 years). Nine of thirty-five (26%) patients underwent one revision surgery between the resection arthroplasty and reimplantation and one of 35 patients (3%) underwent two revision surgeries, which included additional spacer exchange due to wound-related complications and bone grafting procedures because of poor glenoid bone stock. The mean interval between resection arthroplasty and reimplantation was 2.4 months (range: 0.4 to 8 months). In one case, a hemiarthroplasty; in three cases, a total shoulder arthroplasty; and in the remaining 31 cases, a reverse shoulder arthroplasty was performed at the time of reimplantation.

Of the 14 cases that did not undergo reimplantation, infection eradication was achieved in 57% of the cases (8 of 14 cases) after a mean follow-up of 5 years (range: 2.6 to 11 years). Reasons for failure to reimplant were premature death in 5 patients (36%), high general morbidity in 4 patients (29%), satisfaction with the current status in 3 patients (21%), or severe infection with poor bone and soft tissues in 2 patients (14%). Patients who did not undergo subsequent reimplantation were significantly older (76 vs. 67 years, *p* = 0.009), had a significantly higher Charlson comorbidity index (6.2 vs. 3.3, *p* < 0.001), and mortality rate (8/14 vs. 4/35, *p* = 0.002) compared to patients who achieved a successful reimplantation (Table 1). Furthermore, more polymicrobial infections were identified in patients who did not undergo reimplantation. However, this difference was statistically not significant (43% vs. 23%, *p* = 0.18).

A successful infection eradication was achieved in 38 patients of the entire cohort (78%) at the last follow-up. Patients with persistent infection had a significantly higher C-reactive protein on admission (49.4 vs. 14.3 mg/L, *p* = 0.003) and mortality (6/11 vs. 6/38, *p* = 0.02), compared to patients with successful eradication of infection (Table 2).

The overall mortality rate of the entire cohort was 25% (12 of 49 cases) at the latest follow-up, 10% (five cases) within ninety days after resection arthroplasty. Patients who deceased during the follow-up were significantly older (77 vs. 67.3 years, *p* = 0.005) and had a significantly higher Charlson comorbidity index (7.3 vs. 3.1, *p* < 0.001) (Table 3).

## 4. Discussion

Despite the abundance of available literature focusing on two-stage exchange arthroplasty in patients with shoulder PJI, there is a widespread heterogeneity among most studies. The most important fact is the different definition criteria of the shoulder PJI diagnosis, as well as of its treatment success, leading to a lack of comparability. With the expected increase of performed shoulder arthroplasties over the next years, a consensus diagnostic definition and a consensus definition of treatment success for shoulder PJI are getting increasingly relevant and important not only to create a more comparable scientific reporting, but also to diagnose, counsel, and treat patients in a standardized matter.

There is considerable variation in reimplantation rates among studies in literature, ranging from 37% to 97% [7,15,16,17,18,19,20,21]. Only a few studies have dealt with these patients and tried to report on their clinical outcomes and causes for their attrition [7,18,22]. The current study aimed at evaluating all patients who underwent an attempted two-stage exchange arthroplasty for shoulder PJI, irrespective of the subsequent clinical course, and demonstrated that almost one-third of all patients who underwent the first stage of the procedure, did not complete a subsequent reimplantation. Despite having an acceptable infection eradication rate in patients with a permanent spacer, the clinical outcome scores are poor and progressive bone loss can occur with the extended retention of the spacer [18,22]. The current accepted goal of a two-stage exchange arthroplasty protocol is still the reimplantation of a new prothesis to ensure best functional outcome for patients and every effort should be made to improve shoulder function by providing the opportunity for reimplantation.

A variety of reasons can lead to failure to reimplant, including mortality, medical comorbidities, uncontrollable infection leading to amputation of the limb or lifetime antibiotic suppression, and unwillingness of patients to undergo a second surgery, as well as patient’s satisfaction with the current status. Similar to the hip and knee literature [23,24], premature mortality and high general morbidity were the most common causes for not being able to proceed with the intended reimplantation in this study. CCI and older age seemed to be risk factors for failure to reimplant, as the patients in the group without reimplantation had significantly higher CCI and were significantly older compared to patients with reimplantation. Patients with a higher CCI mostly have poorer health as well as a compromised immune status, which can be due to scar tissue and vascular damage. This local and systemic immune failure can massively decrease the minimal infecting dose of bacteria, predisposing to problems with infection eradication [25].

Furthermore, several studies in hip and knee literature were able to show an association of the microorganism type and likelihood of inability to achieve a reimplantation. To our knowledge, there are no existing studies investigating this association in patients with shoulder PJI. Barton et al. found that patients with a polymicrobial infection had a nearly 8-fold greater likelihood of inability to undergo reimplantation compared to patients with monomicrobial or culture-negative infections [23]. Although it did not achieve statistical significance, we were able to identify more polymicrobial infections in patients who did not undergo reimplantation, compared to patients with a successful reimplantation.

The treatment success rate in terms of infection eradication was as high as 86% among patients with reimplantation. This is comparable to the almost 90% infection control rate reported in a recent systematic review and meta-analysis of 30 studies reporting on two-stage exchange arthroplasty in patients with shoulder PJI [6]. However, when taking the patients without reimplantation into consideration, the overall infection eradication rate dropped to 78%, which is lower than most of the studies in the literature dealing with two-stage exchange arthroplasty. The majority of these studies do not encompass patients who do not complete the second stage of an attempted two-stage exchange arthroplasty and exclude them from their treatment success analysis, thereby leading to a possibly overestimated success of this surgical procedure. In addition, the considerably high inflammatory response in many patients of the study cohort may be a further factor affecting our infection eradication rate, as patients with persistent infection had a significantly higher C-reactive protein on admission compared to patients with successful eradication of infection. High virulent microorganisms mostly induce an acute response and much inflammation with the release of cytokines and elevation of CRP, leading to a potentially worse postoperative clinical course [26,27].

Although mortality and morbidity associated with two-stage exchange arthroplasty for hip and knee periprosthetic joint infection has been one of the main research topics [8,23,24], there is a lack of knowledge in shoulder PJI literature. Cancienne et al. recently showed a mortality rate of 2.2% within the first postoperative year in patients undergoing removal of an infected shoulder prosthesis [7]. This is significantly less than the mortality rate reported in the current study, which was 10% within ninety days after resection arthroplasty and 25% at the latest follow-up, which is similar to what has been reported previously in hip and knee literature [23,24,28]. The mortality in five patients, who deceased in the first 90 days after resection arthroplasty, was related to the infection, whereas the other patients died due to other health issues. This indicated that shoulder PJI and its treatment is associated with a high risk of mortality, especially in patients with older age and higher CCI, as shown in our study. In these patients, it may be reasonable to have a detailed discussion with the patient about their likelihood of treatment success, as well as postoperative mortality and consider alternative treatment options, such as long-term antibiotic suppression. Thus, an optimal treatment algorithm should be based on patient-specific general health status, risk factors, and patient expectations.

Only looking from the perspective of infection eradication would lead to overlook a great subset of patients who are too fragile to endure further surgery for reimplantation, decease prematurely, or refuse further surgery because of low functional demand after implant removal. We therefore suggest that the success of two-stage exchange arthroplasty should be accounted from the point of the first stage, rather than following reimplantation, to consider the failures occurring between the stages and better represent the clinical course of two-stage exchange arthroplasty in patients with shoulder PJI [8].

This study has some limitations. Although the patients’ data were longitudinally collected in our database, the retrospective nature of the study may lead to bias. Despite being the largest cohort in literature dealing with this topic, the study may be underpowered, preventing the significant differences between analyzed groups. Furthermore, we did not include any assessment of functional outcomes, which may be seen as a potential weakness. However, the most compelling outcome measures of the current study were the infection eradication, reimplantation, and mortality rates. In addition, there is already an abundance of available literature focusing on the functional outcome of patients after two-stage exchange arthroplasty or antibiotic cement spacer retention. The complexity of our study cohort, due to multiple previous revision surgeries, can furthermore alter our results, making our results to be generalized and difficult to compre with other studies. Finally, infection-related mortality was difficult to confirm in patients who died outside of the hospital and the precise cause of death in these cases could not be determined, which can alter our treatment success rate.

## 5. Conclusions

While the two-stage exchange arthroplasty can lead to high rates of infection eradication, a third of patients never undergo the second stage of the procedure due to a variety of reasons, including premature mortality, high general morbidity, and low functional demand. Furthermore, shoulder PJI and its treatment is associated with a high risk of mortality, especially in patients with older age and higher CCI. This information needs to be accounted for when counseling frail and elderly patients on the chances and risks before undergoing two-stage exchange arthroplasty for shoulder PJI.

## Figures and Tables

**Table 1:** Demographic data, clinical, and laboratory findings of the study cohort and groups with and without subsequent reimplantation.

Variable	All Patients, *n* = 49	Reimplantation Group, *n* = 35	No Reimplantation Group, *n* = 14	*p*-Value ^1^
Mean age, y *	69.7 ± 11	67.1 ± 10.6	76 ± 9.7	0.009
Gender ⧫				
Male	19 (39)	15 (43)	4 (29)	0.5
Female	30 (61)	20 (57)	10 (71)	
CRP at admission (mg/L) *	21.1 ± 32.4	19 ± 36.2	26.8 ± 20	0.46
CCI *	4.1 ± 2.8	3.3 ± 2.1	6.2 ± 3.4	<0.001
Mortality after first stage ⧫				
Ninety days	5 (10)	0 (0)	5 (36)	0.001
Last follow-up	12 (25)	4 (11)	8 (57)	0.002
Polymicrobial shoulder PJI ⧫	14 (29)	8 (23)	6 (43)	0.18
Culture-negative shoulder PJI ⧫	8 (16)	7 (20)	1 (7)	0.4
Infection eradication ⧫	38 (78)	30 (86)	8 (57)	0.06

^1^ Statistical analysis was only undertaken between reimplantation and no reimplantation groups. * The values are given as the mean and the standard deviation. ⧫ The values are given as the number with the percentage of the group in parentheses. CCI—Charlson comorbidity index.

**Table 2:** Demographic data, clinical, and laboratory findings of the groups with infection eradication and infection persistence.

Variable	Infection Eradication *n* = 38	Infection Persistence *n* = 11	*p*-Value
Mean age, y *	69.1 ± 10.7	71 ± 12.4	0.5
CRP at admission (mg/L) *	14.3 ± 17	49.4 ± 60	0.003
CCI *	4 ± 2.8	4.8 ± 3	0.37
Mortality after first stage ⧫			
Ninety days	0 (0)	5 (46)	<0.001
Last follow-up	6 (16)	6 (54)	0.02
Infection subgroups			
Definitive	11 (29)	5 (46)	0.47
Probable	22 (58)	3 (27)	0.1
Possible	5 (13)	3 (27)	0.4
Polymicrobial shoulder PJI ⧫	11 (29)	3 (27)	1.0
Culture-negative shoulder PJI ⧫	5 (13)	3 (27)	0.4

* The values are given as the mean and the standard deviation. ⧫ The values are given as the number with the percentage of the group in parentheses. CCI: Charlson comorbidity index.

**Table 3:** Demographic data, clinical, and laboratory findings of the alive and deceased patients.

Variable	Patients Alive *n* = 37	Patients Deceased, *n* = 12	*p*-Value
Mean age, yr *	67.3 ± 10.6	77 ± 9	0.005
CRP at admission (mg/L) *	17 ± 20.2	34.2 ± 55.6	0.12
CCI *	3.1 ± 1.9	7.3 ± 2.8	<0.001
Polymicrobial shoulder PJI ⧫	12 (29)	2 (27)	0.5
Culture-negative shoulder PJI ⧫	6 (13)	2 (27)	1.0

* The values are given as the mean and the standard deviation. ⧫ The values are given as the number with the percentage of the group in parentheses. CCI. Charlson comorbidity index.

## Data Availability

Data available on request due to restrictions, e.g., privacy or ethical.
